# Data supporting micromechanical models for the estimation of Young's modulus and coefficient of thermal expansion of titanate nanotube/Y_2_W_3_O_12_/HDPE ternary composites

**DOI:** 10.1016/j.dib.2019.104247

**Published:** 2019-07-15

**Authors:** Patricia I. Pontón, Katia Yamada, Marco V. Guamán, Michel B. Johnson, Mary Anne White, Bojan A. Marinkovic

**Affiliations:** a New Materials Laboratory, Department of Materials, Escuela Politécnica Nacional, 170525, Quito, Ecuador; b Centro Universitário de Volta Redonda – UNIFOA, Av. Paulo Erlei Alves Abrantes 1325, Volta Redonda, RJ, Brazil; c Department of Mechanical Engineering, Escuela Politécnica Nacional, 170525, Quito, Ecuador; d Clean Technologies Research Institute, Dalhousie University, Halifax, Nova Scotia, B3H 4R2, Canada; e Department of Chemistry, Dalhousie University, Halifax, Nova Scotia, B3H 4R2, Canada; f Department of Chemical and Materials Engineering, Pontifical Catholic University of Rio de Janeiro, 22451-900, Rio de Janeiro, RJ, Brazil

**Keywords:** Elastic constants, Coefficient of thermal expansion, Micromechanics, Thermomiotics, Titanate nanostructure, Three-phase composites

## Abstract

This article presents several micromechanical models to predict the Young's modulus and the coefficient of thermal expansion of titanate nanotube/Y_2_W_3_O_12_/HDPE composites. The equations and assumptions of the selected micromechanical models are described in detail for this ternary system. Data of the elastic constants, coefficient of thermal expansion of composite components and other associated parameters, obtained either by literature survey or processing of literature information, are compiled in this work. For further interpretation of the data presented in this article, please see our research article entitled “The effect of titanate nanotube/Y_2_W_3_O_12_ hybrid fillers on mechanical and thermal properties of HDPE-based composites” (Pontón et al., 2019).

**Table:** Specifications Table

Subject area	*Ceramics and composites*
More specific subject area	*Polymer-based composites*
Type of data	*Table, figure, scheme*
How data was acquired	*Literature survey*
Data format	*Raw, processed*
Experimental factors	*Data compilation from available literature. Data contain about 23 references.*
Experimental features	*Elastic constants of composite components, as well as, their coefficient of thermal expansion and other parameters, required for the application of the defined micromechanical models, were acquired from the literature or computed from equations presented herein.*
Data source location	*From the literature and author's calculation. The references are specified in the corresponding sections.*
Data accessibility	*Data are accessible in this article.*
Related research article	*These data are related to our recent research article entitled “The effect of titanate nanotube/Y*_*2*_*W*_*3*_*O*_*12*_*hybrid fillers on mechanical and thermal properties of HDPE-based composites” (Pontón* et al.*,**2019*[1]*)*

**Table:** 

**Value of the Data** • The comprehensive data collection of mechanical and thermal properties of the composite components, obtained either by literature review or processing of literature information, are useful for micromechanical modeling of different ternary polymer composites filled with titanate nanotubes and Y_2_W_3_O_12_. • The micromechanical modeling allows the design of ternary polymer composites with specific features, such as reduced thermal expansion and improved stiffness, from extremely low amounts of titanate nanotube/Y_2_W_3_O_12_ hybrid filler and can be extended, in principle, to other polymer matrices or similar particulate hybrid fillers comprising different types of 1-D morphologies. • The optimum titanate nanotube/Y_2_W_3_O_12_ theoretical mass ratio for a specific application can be estimated from micromechanical analysis, depending on the properties expected for composites, as an initial criterion to define the amounts of fillers to be used in the manufacturing of composites. • The approaches suggested in this work for the application of micromechanical models of binary composites to ternary ones, especially for the prediction of the coefficient of thermal expansion by Schapery's model could be considered as a benchmark for further modeling of other ternary polymer composites (reinforced with two different nanofillers), since articles dealing with the prediction of thermal expansion properties of three phase composites are still scarce.

## 1 Data

The data presented in this article include a collection of four micromechanical models to predict the stiffness of polymer composites reinforced with particulate hybrid fillers comprising titanate nanotubes (TTNT) and yttrium tungstate (Y_2_W_3_O_12_), which is a thermomiotic-like filler. These models are: the rule of mixtures (ROM), modified rule of mixtures (MROM), Halpin-Tsai and Hashin-Shtrikman. The equations of these models and the corresponding assumptions (see footnotes) are summarized in Table 1, along with the data of elastic constants and specific parameters for each composite phase. Table 2 presents a compendium of the micromechanical models chosen for the estimation of the coefficient of thermal expansion (CTE) of TTNT/Y_2_W_3_O_12_/HDPE composites, such as the rule of mixtures (ROM), Turner's and Schapery's models. The assumptions of these models (see footnotes) are also described, as well as, the bulk modulus and CTE for each composite phase.

**Table 1:** Models for prediction of Young's modulus of HDPE-based composites reinforced with TTNT/Y_2_W_3_O_12_ hybrid filler and data associated with the elastic constants and specific parameters for each composite phase.

Model	Prediction	Eq.	Variables and parameters
Rule of mixture (ROM)	$E_{c}=E_{f_{1}}\phi_{f_{1}}+E_{f_{2}}\phi_{f_{2}}+E_{m}\left(1-\phi_{f_{1}}-\phi_{f_{2}}\right)$	(1)	$E_{c}$=Young's modulus of the composite
	where,		$E_{m}$^a^=Young's modulus of HDPE=0.824 GPa [1]
	$E_{f_{2}}=\frac{9K_{f_{2}}G_{f_{2}}}{3K_{f_{2}}+G_{f_{2}}}$	(2)	$E_{f_{1}}$^b^=Young's modulus of TTNT∼260 GPa [2]
			$E_{f_{2}}$=Young's modulus of Y_2_W_3_O_12_, calculated from Eq. 2.
			$K_{f_{2}}$=Bulk modulus of Y_2_W_3_O_12_=25 GPa [3]
			$G_{f_{2}}$^c^=Shear modulus of Y_2_W_3_O_12_∼ 12 GPa [4]
			$∴E_{f_{2}}∼31\;GPa$
			$\phi_{f_{1}}$= volume fraction of TTNT
			$\phi_{f_{2}}$= volume fraction of Y_2_W_3_O_12_
Modified rule of mixture (MROM) [5]	$E_{c}=\beta_{1}E_{f_{1}}\phi_{f_{1}}+\beta_{2}E_{f_{2}}\phi_{f_{2}}+E_{m}\left(1-\phi_{f_{1}}-\phi_{f_{2}}\right)$	(3)^d^	β=strengthening factor (also called as modulus efficiency factor), ranging between 0 and 1 [6].
			$\beta_{1}$^e^=strengthening factor of TTNT∼ 0.2 [7], [8]
			$\beta_{2}$^f^ =strengthening factor of Y_2_W_3_O_12_∼0.1
Halpin-Tsai model^g^[9], [10]	$E_{C}=E_{m_{1}}\left[\frac{3}{8}\left(\frac{1+\zeta_{f_{1}}\eta_{L}\phi_{f_{1}}}{1-\eta_{L}\phi_{f_{1}}}\right)+\frac{5}{8}\left(\frac{1+2\eta_{T}\phi_{f_{1}}}{1-\eta_{T}\phi_{f_{1}}}\right)\right]$	(4)	$E_{m_{1}}$^g^ =Young's modulus of HDPE/Y_2_W_3_O_12_ composite, which is considered as a new matrix, calculated with Eq. 5.
	where,		$\zeta_{f_{1}}$= shape parameter of TTNT∼20, calculated with Eq. 7.
	$E_{m_{1}}=\frac{E_{m}\left(1+\zeta_{f_{2}}\eta_{f_{2}}\phi_{f_{2}}\right)}{1-\eta_{f_{2}}\phi_{f_{2}}}$	(5)	$l_{f_{1}}$^h^= length of TTNT ∼ 100 nm
	$\eta_{f_{2}}=\frac{\frac{E_{f_{2}}}{E_{m}}-1}{\frac{E_{f_{2}}}{E_{m}}+\zeta_{f_{2}}}$	(6)	$d_{f_{1}}$= diameter of TTNT ∼ 10 nm [11]
	$\zeta_{f_{1}}=2\left(\frac{l_{f_{1}}}{d_{f_{1}}}\right)$	(7)	$\zeta_{f_{2}}$=shape parameter of Y_2_W_3_O_12_∼ 2, as the first approximation for spherical particulate fillers.
	$\eta_{L}=\frac{\frac{E_{f_{1}}}{E_{m_{1}}}-1}{\frac{E_{f_{1}}}{E_{m_{1}}}+\zeta_{f_{1}}}$	(8)	
	$\eta_{T}=\frac{\frac{E_{f_{1}}}{E_{m_{1}}}-1}{\frac{E_{f_{1}}}{E_{m_{1}}}+2}$	(9)	
Hashin-Shtrikman model^i^[15], [16], [17]	$E_{c}^{l}=\frac{9K_{c}^{l}G_{c}^{l}}{3K_{c}^{l}+G_{c}^{l}}$	(10)	$E_{c}^{l}$, $E_{c}^{u}=$lower and upper bounds of Young's modulus of composite
	$E_{c}^{u}=\frac{9K_{c}^{u}G_{c}^{u}}{3K_{c}^{u}+G_{c}^{u}}$	(11)	$K_{c}^{l}$, $K_{c}^{u}$=lower and upper bounds of bulk modulus of composite calculated from Eqs. 12 and 13.
	where,		$\phi_{hyb}$=volume fraction of the hybrid filler calculated with Eq. 14.
	$K_{c}^{l}=K_{m}+\frac{\phi_{hyb}}{\frac{1}{K_{hyb}-K_{m}}+\frac{3\left(1-\phi_{hyb}\right)}{3K_{m}+4G_{m}}}$	(12)	$K_{hyb}$=bulk modulus of the hybrid filler computed with Eq. 15.
	$K_{c}^{u}=K_{hyb}+\frac{1-\phi_{hyb}}{\frac{1}{K_{m}-K_{hyb}}+\frac{3\phi_{hyb}}{3K_{hyb}+4G_{hyb}}}$	(13)	${K_{f}}_{1}$^j^=bulk modulus of TTNT∼158 GPa [18]
	$\phi_{hyb}=\phi_{f_{1}}+\phi_{f_{2}}$	(14)	$K_{f_{2}}$ =bulk modulus of Y_2_W_3_O_12_=25 GPa [15], [16], [17]
	$K_{hyb}=\phi {'}_{f_{1}}K_{f_{1}}+\phi {'}_{f_{2}}K_{f_{2}}$	(15)	$\phi {'}_{f_{1}}$^k^=volume fraction of TTNT within the hybrid filler calculated with Eq. 16.
	$\phi {'}_{f_{1}}=\frac{\phi_{f_{1}}}{\phi_{f_{1}}+\phi_{f_{2}}}$	(16)	$\phi {'}_{f_{2}}$^k^=volume fraction of Y_2_W_3_O_12_ within the hybrid filler computed with Eq. 17.
	$\phi {'}_{f_{2}}=\frac{\phi_{f_{2}}}{\phi_{f_{1}}+\phi_{f_{2}}}$	(17)	$K_{m}$=bulk modulus of HDPE=0.824 GPa, calculated from Eq. 18.
	$K_{m}=\frac{E_{m}G_{m}}{3\left(3G_{m}-E_{m}\right)}$	(18)	$G_{m}$=shear modulus of HDPE=0.309 GPa, calculated from Eq. 19.
	$G_{m}∼\frac{3}{8}E_{m}$	(19)	$G_{c}^{l}$, $G_{c}^{u\;}$=lower and upper bounds of shear modulus of composite computed with Eq. 20 and Eq. 21.
	$G_{c}^{l}=G_{m}+\phi_{hyb}{\left[\frac{1}{G_{hyb}-G_{m}}+\frac{6\left(1-\phi_{hyb}\right)\left(K_{m}+2G_{m}\right)}{5G_{m}\left(3K_{m}+4G_{m}\right)}\right]}^{-1}$	(20)	$G_{hyb}$ =shear modulus of the hybrid filler calculated with Eq. 22.
	$G_{c}^{u}=G_{hyb}+\left(1-\phi \right){\left[\frac{1}{G_{m}-G_{hyb}}+\frac{6\phi \left(K_{hyb}+2G_{hyb}\right)}{5G_{hyb}\left(3K_{hyb}+4G_{hyb}\right)}\right]}^{-1}$	(21)^m^	$G_{f_{1}}$^n^= shear modulus of TTNT∼ 106.06 GPa, calculated with Eq. 2, but using the corresponding data assumed for TTNT.
	$G_{hyb}=\phi {'}_{f_{1}}G_{f_{1}}+\phi {'}_{f_{2}}G_{f_{2}}$	(22)^m^	$G_{f_{2}}$^c^= shear modulus of Y_2_W_3_O_12_∼ 12 GPa [15], [16], [17].

a Value obtained from tensile test [1].

b Young's modulus of hydrothermally synthesized TTNT are not reported in the literature. Therefore, corresponding value of hydrothermally synthesized titanate nanoribbons [2] was assumed, which is similar to Young's modulus of TiO_2_ ($E_{TiO_{2}}=282.76\;GPa$) [12], [13].

c *G_f2_* is unavailable in the literature. Hence, $G_{Y_{2}Mo_{3}O_{12}}$ was used [14], since bulk moduli of both materials are similar ($K_{Y_{2}Mo_{3}O_{12}}$=21 GPa).

d The product between β and $E_{f}$ is termed as effective Young's modulus of filler ($E_{eff}$).

e Since the length of TTNT is much smaller than the specimen thickness, it is expected that TTNT are randomly oriented in 3D [9], [10]. Thus, $\beta_{1}$ is assumed as 0.2, value used in the literature as a first approximation for 3D randomly oriented carbon nanotubes [9], [10] and particulate fillers, such as TiO_2_[9], [10].

f Value computed from linear fitting of Young's moduli of HDPE/Y_2_W_3_O_12_ composites as a function of Y_2_W_3_O_12_ vol fraction [9], [10] (see Fig. 1 in this article).

g To apply the Halpin-Tsai equation of binary composites reinforced with randomly distributed short fibers for modeling $E_{C}$ of this ternary system, HDPE/Y_2_W_3_O_12_ can be considered as a new matrix reinforced with TTNT, which are treated as randomly oriented short fibers, as suggested by Xiao et al. [9], [10] that used this model for a similar three-phase composite. $E_{m_{1}}$is calculated with Halpin-Tsai equation used for Young's modulus of particulate composites, Eq. 5 in this work.

h The length of TTNT was determined from TEM images [9], [10].

i To apply Hashin-Shtrikman model of binary composites for modeling $E_{C}$ of this ternary system, the hybrid filler (both TTNT and Y_2_W_3_O_12_) is treated as a whole unique dispersed phase, while the continuous phase is HDPE.

j Bulk modulus of hydrothermally synthesized TTNT is not reported in the literature. Hence, corresponding value of analogous anatase TiO_2_ nanotubes, synthesized by a similar alkaline hydrothermal method followed by an acid washing and annealing treatment, was used [15], [16], [17].

k The volume fractions of TTNT and Y_2_W_3_O_12_ within the hybrid filler are presented in Table 3.

m These relationships are applicable when $K_{m}<K_{f}$ and $G_{m}<G_{f}$.

n The shear modulus of TTNT was estimated considering them as isotropic materials as a first approximation.

**Table 2:** Micromechanical models for prediction of CTE of HDPE-based composites reinforced with TTNT/Y_2_W_3_O_12_ hybrid filler and data associated with the elastic constants and CTEs for each composite phase.

Model	Prediction	Eq.	Variables and parameters
Rule of mixture (ROM)	$\alpha_{c}=\alpha_{f_{1}}\phi_{f_{1}}+\alpha_{f_{2}}\phi_{f_{2}}+\alpha_{m}\left(1-\phi_{f_{1}}-\phi_{f_{2}}\right)$	(23)	$\alpha_{c}$=CTE of the composite
			$\alpha_{m}$^a^=CTE of matrix (HDPE)=188x10^-6^ °C^−1^[1]
			$\alpha_{f_{1}}$^b^=CTE of TTNT=9x10^-6^ °C^−1^[19], [20]
			$\alpha_{f_{2}}$=CTE of Y_2_W_3_O_12_= −7.0x10^-6^ °C^−1^[21]
			$\phi_{f_{1}}$=volume fraction of TTNT
			$\phi_{f_{2}}$=volume fraction of Y_2_W_3_O_12_
Turner's model [22]	$\alpha_{c}=\frac{\phi_{f_{1}}K_{f_{1}}\alpha_{f_{1}}+\phi_{f_{2}}K_{f_{2}}\alpha_{f_{2}}+\alpha_{m}\left(1-\phi_{f_{1}}-\phi_{f_{2}}\right)K_{m}}{\phi_{f_{1}}K_{f_{1}}+\phi_{f_{2}}K_{f_{2}}+\left(1-\phi_{f_{1}}-\phi_{f_{2}}\right)K_{m}}$	(24)	${K_{f}}_{1}$^c^=bulk modulus of TTNT∼158 GPa [19], [20]
			$K_{f_{2}}$=bulk modulus of Y_2_W_3_O_12_=25 GPa [3]
			$K_{m}$^d^= bulk modulus of HDPE=0.824 GPa, calculated from Eq. 18.
Schapery's [23] model e	$\alpha_{c}^{l}=\alpha_{m}+\frac{K_{hyb}}{K_{c}^{u}}\frac{\left(K_{m}-K_{c}^{u}\right)\left(\alpha_{hyb}-\alpha_{m}\right)}{\left(K_{m}-K_{hyb}\right)}$	(25)	$\alpha_{c}^{l}$, $\alpha_{c}^{u}=$lower and upper bounds of CTE of the composite reinforced with the hybrid filler.
	$\alpha_{c}^{u}=\alpha_{m}+\frac{K_{hyb}}{K_{c}^{l}}\frac{\left(K_{m}-K_{c}^{l}\right)\left(\alpha_{hyb}-\alpha_{m}\right)}{\left(K_{m}-K_{hyb}\right)}$	(26)	$K_{hyb}$=bulk modulus of the hybrid filler computed with Eq. 15.
	where,		$\alpha_{hyb}$=CTE of the hybrid filler calculated with Eq. 27.
	$\alpha_{hyb}=\phi {'}_{f_{1}}\alpha_{f_{1}}+\phi {'}_{f_{2}}\alpha_{f_{2}}$	(27)	$\phi {'}_{f_{1}}$=volume fraction of TTNT within the hybrid filler calculated with Eq. 16 (see Table 1).
			$\phi {'}_{f_{2}}$=volume fraction of Y_2_W_3_O_12_ within the hybrid filler computed with Eq. 17 (see Table 1)
			$K_{c}^{l}$, $K_{c}^{u\;}$= lower and upper bounds of bulk modulus of composite reinforced with the hybrid filler [16], [17], calculated with Eq. 12 and Eq. 13.

a Value measured by dilatometry in the temperature range of 30–70 °C and during the second heating cycle [1].

b CTE of TTNT is unavailable in the literature. Therefore, corresponding value of TiO_2_ was assumed.

c Bulk modulus of hydrothermally synthesized TTNT is not reported in the literature. Hence, corresponding value of analogous anatase TiO_2_ nanotubes, synthesized by a similar alkaline hydrothermal method followed by an acid washing and annealing treatment, was used [18].

d The bulk modulus of HDPE was calculated using the experimental Young's modulus of HDPE, assuming that this remains unchanged after heating samples up to 70 °C, as a first approximation.

e Since $K_{c}^{l}$ and $K_{c}^{u}$ for ternary phase composites are calculated in the literature using Eq. 12 and 13 [16], [17], and Schapery's model takes into account these two values, it is reasonable to compute $\alpha_{c}^{l}$ and $\alpha_{c}^{u}$ defining $K_{hyb}$ and $\alpha_{hyb}$ by the rule of mixture (see Eq. 15 and Eq. 27, respectively).

## 2 Experimental design, materials, and methods

**Scheme. 1:**
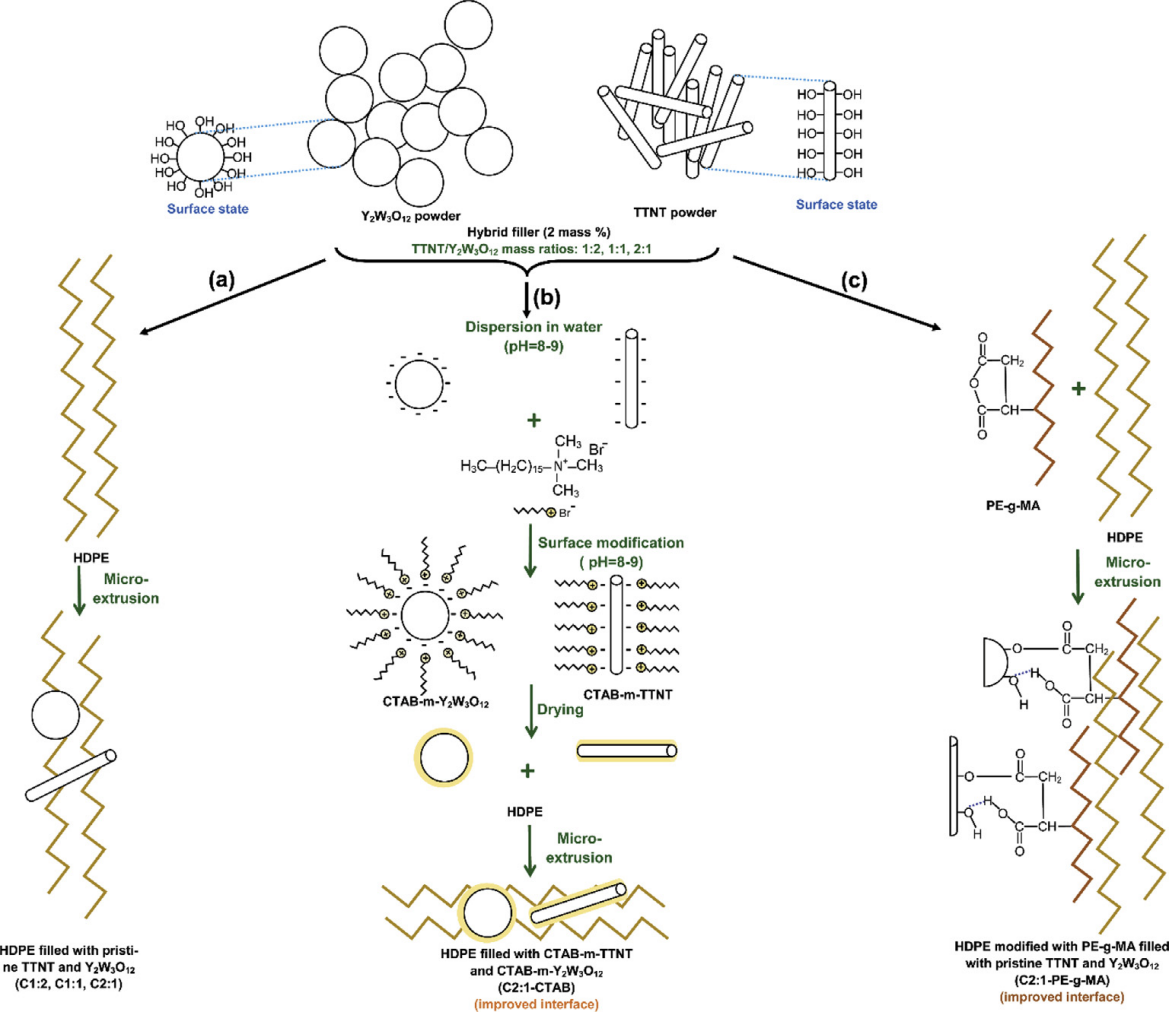
Graphic illustration of the approaches used for the preparation of HDPE-based composites reinforced with (a) pristine hybrid filler, (b) CTAB modified hybrid filler, and (c) pristine hybrid filler with addition of PE-g-MA as compatibilizer (·····representing hydrogen bonding).

The data of micromechanical models presented in Table 1, Table 2 were selected from the literature to describe the TTNT/Y_2_W_3_O_12_/HDPE ternary system, depicted in Scheme 1. These ternary composites can be composed by different TTNT/Y_2_W_3_O_12_ mass ratios: 1:2, 1:1 and 2:1 and prepared using three approaches whether the hybrid filler and the matrix are modified or not: i) pristine hybrid fillers < composites denoted as C1:2, C1:1 and C2:1>, ii) hybrid fillers modified with cetyltrimethylammonium bromide (CTAB) <composites designated as C2:1-CTAB> and iii) HDPE modified with polyethylene-grafted maleic anhydride (PE-*g*-MA) as compatibilizer < composites called as C2:1-PE-g-MA>.

The computation of the volume fractions of TTNT and Y_2_W_3_O_12_ ($\phi_{f_{1}}$ and $\phi_{f_{2}}$ , respectively) for the application of these models is presented in reference [1]. The mechanical and thermal properties of TTNT are assumed to be equal to those values of similar titania-based materials, as a first approximation, since they are not reported in the literature.

For the application of MROM for the TTNT/Y_2_W_3_O_12_/HDPE ternary system (see Eq. 3), the strengthening factor of Y_2_W_3_O_12_ ($\beta_{2}$) was calculated from the experimental Young's moduli of HDPE/Y_2_W_3_O_12_ composites using data reported by Pontón et al. [14]. The MROM for these binary composites can be expressed as:

(28) $$E_{c_{2}}=\beta_{f_{2}}E_{f_{2}}\phi_{f_{2}}+E_{m}\left(1-\phi_{f_{2}}\right)$$

(29) $$E_{c_{2}}=\left(\beta_{f_{2}}E_{f_{2}}-E_{m}\right)\phi_{f_{2}}+E_{m}$$

Therefore, $\beta_{2}$ can be calculated from the slope of $E_{c_{2}}$ as a function of $\phi_{f_{2}}$. The linear fitting of experimental Young's moduli of HDPE/Y_2_W_3_O_12_ composites is presented in Fig. 1.

**Fig. 1:**
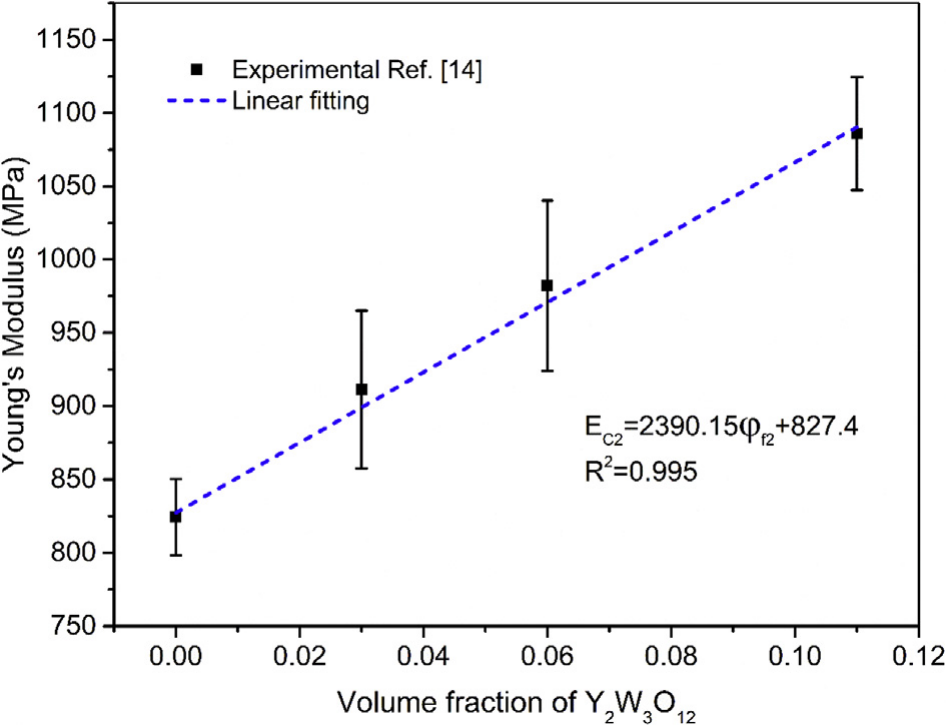
Experimental Young's modulus ($E_{c_{2}}$) of HDPE/Y_2_W_3_O_12_ composites as a function of volume fraction of Y_2_W_3_O_12_[14] and linear fitting of experimental data. Hence, $\beta_{2}$ is calculated as follows , using Eqs. (30) and (31):(30)$$E_{c_{2}}=2390.15\phi_{f_{2}}+827.4\;\left(R^{2}=0.995\right)$$(31)$$\beta_{f_{2}}E_{f_{2}}-E_{m}=2390.15$$$$∴\beta_{f_{2}}=0.1$$

The volume fractions of TTNT and Y_2_W_3_O_12_ within the hybrid filler, $\phi {'}_{f_{1}}$ and $\phi {'}_{f_{2}}$, respectively, are presented in Table 3. These values were calculated with Eq. 16 and Eq. 17 from the volume fractions of both fillers ($\phi_{f_{1}}$, $\phi_{f_{2}}$) inside the whole composite, and they are required for application of both Hashin-Shtrikman and Schapery's models.

**Table 3:** Volume fractions of TTNT and Y_2_W_3_O_12_ within the hybrid filler.

Nomenclature of composite sample^a^	Volume fraction		Volume fraction of hybrid filler ($\phi_{\mathrm{hyb}}$)	Volume fraction within the filler		Hybrid filler
	TTNT ($\phi_{f_{1}}$)	Y_2_W_3_O_12_ ($\phi_{f_{2}}$)		TTNT ($\phi {'}_{f_{1}}$)	Y_2_W_3_O_12_ ($\phi {'}_{f_{2}}$)	
C1:2	0.0021	0.0028	0.0049	0.43	0.57	
C1:1	0.0031	0.0021	0.0052	0.60	0.40	
C2:1	0.0042	0.0014	0.0056	0.75	0.25	
C2:1(CTAB)	0.0042	0.0014	0.0056	0.75	0.25	
C2:1(PE-g-MA)	0.0042	0.0014	0.0056	0.75	0.25	

a Nomenclature of composite samples in accordance to Scheme 1.

There is a lack of information in the literature related to the application of Schapery's model to predict the CTE of polymer composites reinforced with a hybrid filler. The present approach of application of Schapery's model equation for binary composites to ternary ones (see Eq. 25 and Eq. 26) was based on the assumptions previously used in Hashin-Shtrikman model (see footnote “i” in Table 1), since both models depend on $K_{c}^{l}$ and $K_{c}^{u\;}$.

All micromechanical models presented in this article assumes perfect interfaces and homogenous dispersion of hybrid fillers. Experimental deviations from these models can be observed as a result of different dispersion states of fillers inside the matrix and/or the presence of the interfacial groups at hybrid filler-matrix interfaces.
